# Impact of Educational Level on Study Attrition and Evaluation of Web-Based Computer-Tailored Interventions: Results From Seven Randomized Controlled Trials

**DOI:** 10.2196/jmir.4941

**Published:** 2015-10-07

**Authors:** Dominique A Reinwand, Rik Crutzen, Iman Elfeddali, Francine Schneider, Daniela Nadine Schulz, Eline Suzanne Smit, Nicola Esther Stanczyk, Huibert Tange, Viola Voncken-Brewster, Michel Jean Louis Walthouwer, Ciska Hoving, Hein de Vries

**Affiliations:** ^1^ CAPHRI Department of Health Promotion Maastricht University Maastricht Netherlands; ^2^ Jacobs Center for Lifelong Learning and Institutional Development Jacobs University Bremen Germany; ^3^ GGz Breburg Tilburg Netherlands; ^4^ Tranzo Department Tilburg University Tilburg Netherlands; ^5^ Amsterdam School of Communication Research (ASCoR) Department of Communication Science University of Amsterdam Amsterdam Netherlands; ^6^ CAPHRI Department of Family Medicine Maastricht University Maastricht Netherlands

**Keywords:** dropout, attrition, educational level, computer tailoring, Web-based intervention, eHealth, evaluation, meta-analysis

## Abstract

**Background:**

Web-based computer-tailored interventions have shown to be effective in improving health behavior; however, high dropout attrition is a major issue in these interventions.

**Objective:**

The aim of this study is to assess whether people with a lower educational level drop out from studies more frequently compared to people with a higher educational level and to what extent this depends on evaluation of these interventions.

**Methods:**

Data from 7 randomized controlled trials of Web-based computer-tailored interventions were used to investigate dropout rates among participants with different educational levels. To be able to compare higher and lower educated participants, intervention evaluation was assessed by pooling data from these studies. Logistic regression analysis was used to assess whether intervention evaluation predicted dropout at follow-up measurements.

**Results:**

In 3 studies, we found a higher study dropout attrition rate among participants with a lower educational level, whereas in 2 studies we found that middle educated participants had a higher dropout attrition rate compared to highly educated participants. In 4 studies, no such significant difference was found. Three of 7 studies showed that participants with a lower or middle educational level evaluated the interventions significantly better than highly educated participants (“Alcohol-Everything within the Limit”: *F*_2,376_=5.97, *P*=.003; “My Healthy Behavior”: *F*_2,359_=5.52, *P*=.004; “Master Your Breath”: *F*_2,317_=3.17, *P*=.04). One study found lower intervention evaluation by lower educated participants compared to participants with a middle educational level (“Weight in Balance”: *F*_2,37_=3.17, *P*=.05). Low evaluation of the interventions was not a significant predictor of dropout at a later follow-up measurement in any of the studies.

**Conclusions:**

Dropout attrition rates were higher among participants with a lower or middle educational level compared with highly educated participants. Although lower educated participants evaluated the interventions better in approximately half of the studies, evaluation did not predict dropout attrition. Further research is needed to find other explanations for high dropout rates among lower educated participants.

## Introduction

Previous studies have demonstrated that Web-based computer-tailored interventions can be effective in motivating individuals to adopt different health behaviors [1-3], such as increasing physical activity [4-10], improving healthy nutrition [11-14], smoking cessation [15-18], and reducing alcohol intake [19-21], and they have been successfully applied to multiple health behaviors [22,23]. In addition, these interventions have been found to be more cost-effective than usual care or nontailored information [24-27].

According to Eysenbach [28], dropout, either not completing the study or missing follow-up measurements, is a “fundamental characteristic” of Internet interventions and a problematic issue. The loss of participants to follow-up, dropout attrition, makes analyses and statements of the effectiveness of these interventions more complicated and less valid [28] because most outcome measures are assessed during follow-up and dropout from the intervention seems to be related to dropout attrition [29]. Therefore, it is important to find out why participants do not complete Web-based studies to ultimately understand and reduce this problem.

Acquiring follow-up measurements from at-risk groups, such as people with a lower educational level, is important because unhealthy lifestyle behaviors are most common among people with a lower educational level [30-32]. Lower educated people are known to eat less fruits and vegetables [33,34], are less physically active [30,35], consume alcohol more often in unhealthy quantities [36], use more tobacco [37-39], and have a higher likelihood of being obese [40] compared to highly educated people. It is not only important to reach this group with Web-based computer-tailored interventions, but also to prevent lower educated participants from dropping out of the follow-up measurements to be able to collect information about the effectiveness of the intervention [29].

Christensen and Mackinnon [41] already raised the issue of insufficient research regarding study dropout in 2006. Since then, findings about dropout among participants with different educational levels are still rarely reported and show ambiguous results. Although some studies revealed that people with a lower educational level have higher dropout rates in Web-based computer-tailored interventions than highly educated people [42-46], other studies did not find educational differences in terms of dropout [46-49]. To the best of our knowledge, no study indicates a significantly higher dropout among highly educated participants, but there remains a need to obtain more insight into dropout among people with different educational levels to be able to reduce dropout.

The reason for dropout among lower educated people is discussed rarely in the literature. Possible reasons for dropout can be intervention characteristics (eg, workload, content), personal characteristics such as educational level, or it can be related to participants’ perceptions of the interventions, such as a lack of perceived benefit, which may result in dissatisfaction [50]. Dissatisfaction with the intervention can be reflected in the evaluation of the intervention. It has been shown that participants who do evaluate the intervention as less positive are more likely to drop out [51] and, therefore, might not be interested in attending follow-up measurements. In other words, evaluation might be a predictor of dropout attrition in Web-based computer-tailored interventions, but little thought has been given to this aspect, which makes it difficult to draw valid conclusions [52].

Therefore, the aim of this study is first to examine if the dropout attrition rates in our 7 randomized controlled trials (RCTs) of Web-based computer-tailored interventions were higher for people with a lower educational level than people with a middle or high educational level. Second, we assessed whether people with different educational levels evaluated these interventions differently. Finally, we analyzed whether participations’ evaluation of the interventions predicted dropout at subsequent follow-up measurements.

## Methods

### Studies

To examine differences in dropout attrition and evaluation between participants with different educational levels, we used a convenience sample of participants from 7 Web-based computer-tailored intervention studies that were conducted in the past years (2010-2014) at the Department of Health Promotion of Maastricht University in the Netherlands.

The studies were RCTs to evaluate interventions that used computer-tailored techniques to improve diverse health behaviors. The study “Master Your Breath” (MYB) focused on increasing physical activity and smoking cessation among chronic obstructive pulmonary disease (COPD) patients and people at risk for COPD. The 3 studies “Stay Quit for You” (SQ4U), “Support to Quit” (STQ), and “Personal Advice in Stopping smoking” (PAS) focused on smoking cessation. “Weight in Balance” (WIB) aimed to prevent obesity by targeting physical activity and energy intake. The study “My Healthy Behavior” (MHB) targeted the following health behaviors: physical activity, fruit and vegetable consumption, alcohol intake, and smoking. The study “Alcohol-Everything within the Limits” (AEL) focused on moderate alcohol intake and is the only study that was not carried out in the Netherlands but in Germany.

All selected studies made use of the I-Change model [53,54], which postulates that the behavior change process has at least 3 phases: awareness, motivation, and action. The first factor is determined by factors such as behavioral awareness, knowledge, and risk perceptions. The second phase is determined by attitudes, social influence beliefs, and self-efficacy expectations, and results in a certain intention to perform a particular behavior. The third factor is determined by self-efficacy, action planning, skills, and barriers. The tailored feedback messages of the studies included in this paper have a strong focus on inter alia these determinants. A detailed description of these RCTs and the related publications can be found in Table 1.

**Table 1:** Summary of the Web-based computer-tailored interventions.

Study	Reference	Participants	Study groups	Intervention	Follow-up
AEL	Design and effects: [20]	German general population aged 18-69 years	Two intervention groups that differed in the computer-tailored feedback strategies (alternating vs summative) compared to 1 control group that received no computer-tailored feedback.	A 3-session, Web-based computer-tailored intervention aiming to reduce alcohol intake in high-risk adult drinkers.	T1=3 months; T2=6 months
MHB	Study protocol: [55]; effects: [20,26]	Dutch general population aged 19-65 years	Two experimental groups (ie, a sequential behavior tailoring condition and a simultaneous behavior tailoring condition) and 1 control group that that received only a tailored health risk appraisal but no motivational computer-tailored feedback.	Five lifestyle behaviors of smoking, alcohol intake, fruit consumption, vegetable consumption, and physical activity addressing computer-tailored feedback at several times.	T1=12 months; T2=24 months
MYB	Study protocol: [56]; effects: [57]	People with or at risk for COPD in the Netherlands	One intervention group received Web-based computer-tailored self-management intervention; the control group received usual care.	Web-based, computer-tailored self-management intervention with the aim to increase physical activity and support smoking cessation.	T1=6 months
PAS	Study protocol: [58]; effects: [18,25,59]	Adult Dutch smokers with intention to stop smoking within 6 months	Intervention group with computer-tailored information to quit smoking compared to control group that received no computer-tailored feedback.	A Web-based computer-tailored smoking cessation intervention.	T1=6 weeks; T2=6 months; T3=12 months
STQ	Study protocol: [60]; effects: [61,62]	Dutch smokers who were motivated to stop smoking and aged ≥18 years	Intervention groups 2 (video/text) × (low/middle/high socioeconomic status). Respondents were assigned to 1 of the intervention groups (text- vs video-tailored feedback) or to the control group (nontailored generic advice).	Comparing Web-based text and a Web-based video-driven computer-tailored approach for low and high SES smokers, this incorporates multiple computer-tailored feedback moments with the aim to support smoking cessation.	T1=6 months; T2=12 months
SQ4U	Study protocol: [63]; effects: [15]	Dutch daily smokers aged 18-65 years who were motivated to stop smoking	Two intervention groups (Action Plan, Action Plan+), 1 control group that received no computer-tailored feedback.	Two computer-tailored interventions to prevent smoking relapse. Provides tailored feedback in the Action Plan+ group after stop smoking attempts, in the Action Plan group after T0 measurement.	T1=6 months; T2=12 months
WIB	Study protocol: [64]; effects: [65]	Normal and overweight adults from the Netherlands	Two intervention groups (video and text) and 1 waiting list control group.	Computer-tailored feedback via text or video to prevent weight gain or support modest weight loss by targeting physical activity and energy intake.	T1=6 months

### Measurement

In all 7 studies, educational level was assessed by asking participants about their highest completed level of education. In-line with national guidelines, educational level was categorized into 3 groups: lower (1=no education, primary or lower vocational school), middle (2=secondary vocational school or high school), and higher (3=higher professional education or university) educational level [66].

All studies included a process evaluation assessment to evaluate the intervention among participants within the intervention group. Participants were asked to evaluate the tailored feedback and the intervention. The process evaluation assessments of the 7 studies included had one item in common that asked participants to grade the intervention that they participated in: “Please evaluate the intervention with a school grade from 1 to 10” (10=highest grade, 1=lowest grade according to the Dutch school grading system; AEL: 15=highest grade, 1=lowest grade, which is in-line with the German school grading system).

To assess dropout attrition, participants who completed the baseline measurement but did not complete the follow-up measurement were characterized as dropouts (1=dropout; 0=completed follow-up). We assessed dropout attrition within differently educated participants for each follow-up measurement separately. Furthermore, we used the last available evaluation moment as predictor of dropout for the following measurement. Table 1 gives an overview of the specific follow-up moments per study.

### Statistical Analysis

All analyses were performed with SPSS 20.0 (IBM Corp, Armonk, NY, USA). Descriptive statistics were used to describe sample characteristics. Per study, a logistic regression analysis was conducted to examine if dropout rates differed for each educational level. To control for multiple testing, the Benjamini and Hochberg linear step-up method was used for each study [67,68]. With the use of an Excel template, the adjusted significance levels were calculated [69].

Differences between the educational levels with regard to evaluation of the Web-based computer-tailored interventions were analyzed by means of ANOVAs and Tukey honestly significant difference (HSD) tests. Control groups were excluded from analysis with regard to evaluation of the intervention because they could not evaluate it.

To be able to give a more general picture of whether lower and higher educated participants from the intervention groups evaluated Web-based computer-tailored interventions differently, the Exploratory Software for Confidence Intervals (ESCI) Excel template [70] was used for pooling the data by means of a meta-analysis (Table 1). The meta-analysis used a random effect model and gave an impression of the overall differences for intervention evaluation between lower and higher educated participants (ie, by subtracting the evaluation of the most different groups, the lower educated participants from the higher educated participants). In one study (MHB), the evaluation item was assessed at multiple follow-up measurements; in this case, we included only the last follow-up measurement [71].

Finally, logistic regression analyses were conducted to examine if dropout was predicted by evaluation in 4 of the 7 interventions among participants with different educational levels. We excluded the studies WIB, PAS, and MYB from this analysis because their evaluation assessment took place during the last follow-up measurement; therefore, it was not possible to assess evaluation as a predictor of dropout in these studies. To identify possible interaction effects, an interaction term of educational level and evaluation was used in the regression model. If this interaction term was significant, then analyses were conducted separately per educational level. Analyses were corrected for age and gender. A *P* value of .05 was used as the significance level for all analysis.

## Results

### Participants

Table 2 shows the educational level, mean age, and gender distribution of the participants within the 7 selected studies at baseline.

**Table 2:** Baseline sample characteristics of the participants in the Web-based computer-tailored interventions.

Study	N	Educational level, n (%)^a^			Age (years), mean (SD)	Gender (male), n (%)
		Low	Middle	High		
AEL	1149	483 (44.8)	256 (23.8)	338 (31.4)	43.82 (15.51)	550 (47.9)
MHB	5055	515 (10.4)	2334 (47.1)	2112 (42.6)	44.15 (12.67)	2661 (52.6)
MYB	1307	386 (29.5)	427 (32.7)	494 (37.8)	57.64 (7.22)	627 (47.9)
PAS	1123	238 (21.2)	513 (45.7)	372 (33.1)	49.47 (32.55)	535 (47.6)
STQ	2099	707 (33.6)	782 (37.3)	612 (29.2)	45.33 (13.21)	821 (39.1)
SQ4U	2031	207 (10.2)	1130 (55.6)	694 (34.2)	40.88 (11.80)	766 (37.7)
WIB	1419	214 (15.1)	436 (30.7)	769 (54.2)	48.13 (11.52)	588 (41.4)

^a^ For reference, the average educational level in Germany for low, middle, and high is 39, 22, and 27, respectively [72]; for the Netherlands, it is 30, 28, and 42, respectively [73].

### Dropout

Table 3 shows the results of the dropout analyses with regard to the educational level for each study, each follow-up measurement including the dropout rates, and study group in detail with high education as the reference group.

**Table 3:** Results of a logistic regression examine dropout attrition among different educational groups.

Study, follow-up, and group			Dropout, n (%)	Educational level^a^			
				Low		Middle	
				OR (95% CI)	*P*	OR (95% CI)	*P*
**AEL**							
	**T1**		398 (34.6)				
		Sequential		0.61 (0.10-3.59)	.58	1.03 (0.15-7.18)	.97
		Simultaneously		1.65 (0.61-4.58)	.32	—^c^	.99
		Control		—^c^	.99	—^c^	.99
	**T2**		436 (37.9)				
		Sequential		1.16 (0.24-5.52)	.89	1.90 (0.18-19.37)	.58
		Simultaneously		1.15 (0.34-3.84)	.81	1.23 (0.21-7.13)	.81
		Control		0.90 (0.11-7.06)	.92	0.51 (0.37-7.09)	.61
**MHB**							
	**T1**		3317 (65.6)				
		Sequential		1.52 (1.14-2.01)	.004^b^	1.05 (0.83-1.32)	.68
		Simultaneously		1.57 (1.18-2.08)	.002^b^	1.39 (1.09-1.78)	.007^b^
		Control		1.32 (1.00-1.73)	.04	1.27 (1.00-1.61)	.04
	**T2**		3602 (71.3)				
		Sequential		1.43 (1.06-1.94)	.01^b^	0.95 (0.74-1.23)	.73
		Simultaneously		1.51 (1.12-2.04)	.006^b^	1.50 (1.16-1.94)	.002^b^
		Control		1.23 (0.93-1.62)	.14	0.97 (0.76-1.23)	.81
**MYB**							
	**T1**		254 (19.4)				
		Intervention		1.33 (0.84-2.12)	.21	1.40 (0.89-2.20)	.13
		Control		1.14 (0.67-1.95)	.61	1.17 (0.70-1.96)	.53
**PAS**							
	**T1**		674 (60.0)				
		Control		0.93 (0.58-1.49)	.77	0.88 (0.59-1.31)	.55
		Tailoring only		2.02 (1.23-3.33)	.005	1.37 (0.93-2.01)	.10
	**T2**		831 (74.0)				
		Control		0.93 (0.54-1.56)	.77	0.89 (0.57-1.39)	.61
		Tailoring only		2.04 (1.15-3.60)	.01	1.41 (0.93-2.15)	.10
	**T3**		967 (86.1)				
		Control		1.42 (0.71-2.86)	.32	0.89 (0.52-1.52)	.67
		Tailoring only		1.41 (0.67-2.97)	.35	1.03 (0.60-1.78)	.90
**STQ**							
	**T1**		1306 (62.2)				
		Video		1.90 (1.24-2.90)	.003^b^	1.39 (0.93-2.09)	10
		Text		1.29 (0.87-1.91)	.19	1.22 (0.83-1.78)	.29
		Control		0.98 (0.67-1.47)	.98	0.71 (0.49-1.05)	.09
	**T2**		1437 (68.5)				
		Video		1.95 (1.26-3.02)	.003^b^	1.39 (0.92-2.09)	.11
		Text		2.31 (1.52-3.51)	.<001^b^	1.29 (0.88-1.89)	.18
		Control		1.36 (0.90-2.04)	.13	1.24 (0.84-1.84)	.66
**SQ4U**							
	**T1**		1251 (61.9)				
		Action Plan		1.26 (0.75-2.12)	.36	1.03 (0.74-1.44)	.83
		Action Plan +		1.71 (0.92-3.18)	.08	1.14 (0.81-1.60)	.44
		Control		1.73 (0.91-3.27)	.09	0.90 (0.63-1.28)	.57
	**T2**		1465 (72.1)				
		Action Plan		2.33 (1.24-4.35)	.01	1.30 (0.91-1.86)	.14
		Action Plan +		2.25 (1-11-4.52)	.02	1.55 (1.07-2.24)	.01
		Control		2.00 (1.02-3.92)	.04	1.35 (0.94-1.94)	.09
**WIB**							
	**T1**		404 (28.5)				
		Video		1.50 (0.87-2.59)	.15	1.23 (0.81-2.01)	.29
		Text		2.29 (1.33-3.95)	.003^b^	1.12 (0.74-1.69)	.60
		Control		1.57 (0.81-3.04)	.18	2.01 (1.22-3.32)	.006^b^

^a^ All analysis are corrected for age and gender. High education is the reference group.

^b^ Significant *P* values after correction for multiple comparisons according to Benjamini-Hochberg.

^c^ Odds ratios are not reported due to low cell count.

After correction for multiple testing, significantly higher dropout rates were found within 3 studies (MHB, STQ, WIB) among lower educated participants compared to higher educated ones. Furthermore, in these 3 studies, dropout attrition was also significantly higher among middle educated participants in comparison with higher educated participants. In 4 of 7 studies (AEL, MYB, PAS, SQ4U), no difference in dropout with regard to educational level was found.

### Evaluation

Table 4 presents differences between the educational groups with regard to evaluation of the Web-based computer-tailored interventions in detail. In 3 of 7 studies (AEL, MHB, MYB), lower educated participants evaluated the intervention significantly higher compared to their counterparts. In one study (WIB), lower educated participants evaluated the intervention less positively compared to middle educated participants.

**Table 4:** Evaluation of the 7 Web-based computer-tailored interventions by different educational levels.

Study and group^a^			Level of education, mean (SD)			*F* (*df1,df2*)	*P*	Tukey HSD, *P*		
			Low	Middle	High			L-M^b^	L-H	M-H
**AEL**										
	**T0**									
		Sequential	11.20 (3.48)	11.12 (3.00)	10.72 (3.62)	0.71 (2,340)	.56	.97	.47	.70
		Simultaneously	11.75 (3.37)	11.49 (2.85)	10.40 (3.61)	5.97 (2,376)	.003	.82	.002	.05
	**T2**									
		Sequential	11.32 (4.27)	11.27 (3.69)	10.39 (4.33)	0.58 (2,229)	.56	.77	.89	.53
		Simultaneously	11.09 (4.28)	11.53 (3.30)	10.81 (3.89)	1.26 (2,246)	.56	.99	.29	.48
**MHB**										
	**T1**									
		Sequential	7.43 (1.08)	7.14 (1.79)	7.03 (1.13)	2.12 (2,201)	.12	.98	.48	.11
		Simultaneously	7.80 (0.91)	6.94 (1.70)	6.56 (2.41)	1.30 (2,178)	.27	.60	.27	.62
	**T2**									
		Sequential	7.78 (1.27)	7.59 (0.94)	7.53 (0.91)	1.04 (2,367)	.35	.64	.37	.71
		Simultaneously	7.94 (0.89)	7.68 (0.92)	7.43 (1.02)	5.52 (2,359)	.004	.30	.01	.05
**MYB**										
	**T1**									
		Intervention	7.07 (1.50)	6.93 (1.23)	6.60 (1.77)	3.17 (2,317)	.04	.77	.05	.17
**PAS**										
	**T3**									
		Tailoring only	6.09 (1.70)	6.72 (1.25)	7.03 (1.20)	2.42 (2,81)	.09	.92	.96	.63
**STQ**										
	**T2**									
		Video	6.48 (1.97)	6.18 (2.17)	6.28 (1.64)	0.38 (2,193)	.69	.67	.83	.95
		Text	6.53 (1.84)	6.51 (1.23)	5.96 (1.72)	3.29 (2,234)	.04	.99	.08	.07
**SQ4U**										
	**T1**									
		Action Plan	6.56 (1.74)	6.63 (1.41)	6.20 (1.60)	1.29 (2,134)	.28	.98	.79	.25
		Action Plan+	6.27 (2.10)	6.49 (1.69)	6.51 (1.27)	0.10 (2,108)	.90	.97	.85	.99
**WIB**										
	**T1**									
		Video	6.99 (1.23)	7.56 (0.78)	7.36 (1.08)	3.17 (2,37)	.05	.05	.27	.21
		Text	6.79 (0.92)	7.32 (0.82)	7.11 (1.24)	0.66 (2,51)	.52	.49	.67	.88

^a^ T specifies the time of the evaluation measurement.

^b^ Level of education: L=low, M=middle, H=high.

**Figure 1:**
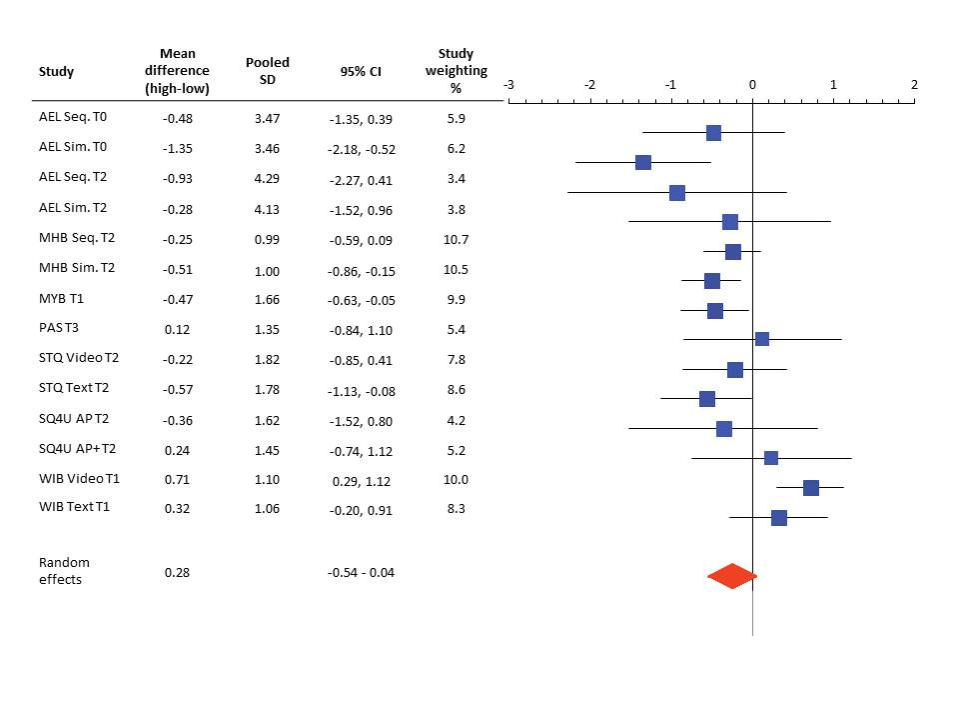
Forest plot of mean differences by random effect model of evaluation of Web-based computer-tailored interventions between highly and lower educated participants. Random effects represent the combined effect.

Furthermore, the meta-analysis of the 7 studies comparing evaluation of lower and higher educated participants indicated that participants with a lower educational level evaluated the interventions significantly more positively compared to highly educated participants (OR 0.28, 95% CI –0.54 to 0.04, *P<*.001) (see Figure 1). Nevertheless, the meta-analysis revealed presence of a moderate level of heterogeneity (*I* ^2^=66.10%) [74,75], which indicates variation across the studies.

### Association of Education and Evaluation with Dropout Attrition

For the 4 studies that evaluated the intervention before the follow-up measurements, a significant interaction between education and evaluation regarding dropout at follow-up was not found (Table 5). Only within the MHB study was a positive association found between the intervention evaluation and educational level. Participants with a middle educational level were more likely to dropout than participants with a higher educational level.

**Table 5:** Association of education and evaluation with dropout attrition at follow-up.

Study and variables^a^		β	*P*	OR (95% CI)	χ^2^_7_	*R* ^2^
**AEL T1**					9.0	.054
	Education low	–1.02	.35	0.35 (0.04-3.14)		
	Education middle	–0.08	.96	0.92 (0.02-37.18)		
	Evaluation T0	0.03	.69	1.03 (0.88-1.19)		
	Education × evaluation		.48			
**MHB T1**					96.9	.053
	Education low	1.90	.05	6.71 (0.97-46.43)		
	Education middle	0.93	.07	2.55 (0.89-7.27)		
	Evaluation T0	–0.01	.84	0.99 (0.90-1.08)		
	Education × evaluation		.18			
**STQ T2**					16.6	.033
	Education low	0.62	.29	1.86 (0.57-6.00)		
	Education middle	0.28	.64	1.32 (0.40-4.35)		
	Evaluation T1	–0.49	.52	0.95 (0.81-1.10)		
	Education × evaluation		.99			
**SQ4U T2**					5.6	.051
	Education low	4.33	.11	76.36 (0.34-16.713.27)		
	Education middle	1.32	.55	3.75 (0.46-303.55)		
	Evaluation T1	0.11	.69	1.11 (0.64-1.92)		
	Education × evaluation		.40			

^a^ T indicates follow-up; high education is reference group. All analyses are corrected for age and gender.

## Discussion

### Dropout Attrition

The first aim of this study was to evaluate whether participants with a lower educational level have higher dropout attrition from Web-based computer-tailored studies than participants with a medium or high educational level. In 3 of these studies, lower and middle educated participants dropped out more frequently compared to higher educated participants.

A possible explanation for the higher dropout rates may be that lower educated participants tend to use written health information more often [76] and spend less time online seeking health information [77,78]. It could be possible that they lose interest in the intervention sooner, which causes them to drop out of the study.

Also, the fact that lower educated people have an unhealthier lifestyle [30-32] might play a role in dropout. Due to tailoring, participants with an unhealthier lifestyle in multiple health behavior interventions received more recommendations to change their health behavior(s) and this has been found to decrease participants’ motivation to change [79]. It might be possible that lower educated participants started the intervention with the aim to change their health behavior, but that receiving information about extensive required changes decreased their self-efficacy to be able to change [80] and could subsequently have decreased their motivation to participate. Another explanation could be that lower educated participants might have been less likely to change their behavior and, thus, may have perceived the recommendations as less feasible, which caused them to drop out of the study [28,81,82]. This could have caused not only usage and nonusage attrition, but also dropout attrition because these 2 kinds of attrition seem to be related [46].

Moreover, lower educated people might be less familiar with Web-based computer-tailored interventions [83,84] and that might result in lower confidence in the effectiveness of those interventions (ie, lower perceived efficacy) and, in turn, could cause an increase in dropout [85,86]. Although these are reasons for nonusage attrition (not using the intervention), it seems convincing that this correlates with dropout attrition because participants who did not evaluate the intervention positively might have little interest in completing follow-up measurements [51].

All participants were asked to complete long questionnaires and received tailored feedback, which must be cognitively processed and requires intensive cognitive performance. Lower educated adults have been shown to have a lower level of health literacy [84,87,88]. They have more difficulties processing new information and this could cause ego depletion [89,90]. Ego depletion may, in turn, reduce the willingness to participate any longer within the study.

Some studies found that dropout attrition could be increased by sending reminders and prompts [78,91-93]. Further research is necessary to evaluate if this is also effective for people with different educational levels.

### Evaluation

Against our initial expectation that lower educated participants might evaluate the interventions less positively, we found that lower educated participants evaluated the intervention in 3 of 7 studies more positively compared to their higher educated counterparts. This might be explained by the finding that highly educated people make more use of the Internet as health information resource, whereas these interventions might be newer and more interesting for lower educated people. A review supported this assumption because it shows that people with a high educational level may make more intensive use of several sources (eg, people form their social network, mass media, health professionals) to gain health-related information compared to lower educated people and they might read the received information more superficially [94]. This might result in less elaboration of the messages and a lower evaluation regarding the novelty of the messages. Due to the use of several resources, highly educated participants also rely less on online information and have lower levels of trust in them, which may negatively influence their evaluation of the intervention [95].

Evaluation was not a significant predictor of dropout at follow-up in any of our studies. This suggests that other factors must be important in explaining why participants did not return to the study for follow-up questionnaires. Dropout analysis performed within these studies has shown that a lower educational level, unhealthy lifestyle, low intention to change the behavior, and low self-efficacy were predictors of dropout [20,96,97].

### Limitations and Strengths

First, the only item all studies had in common concerning the evaluation of the interventions was an overall grade participants assigned to the intervention. Although we can assume that this item gives an overall impression of participants’ evaluation, it might be that participants with different educational levels liked and disliked different aspects of the intervention (eg, layout, provided information, or personal relevance), which was not reflected in this overall grade. However, the evaluation of these different aspects was not equally assessed in all 7 studies. Furthermore, it is possible that participants who did not like the intervention dropped out before completing the evaluation item. In this study, we included only those participants that completed the evaluation item and assessed follow-up at the subsequent measurement.

Second, all interventions were based on the I-Change model and targeted the same social cognitive determinants to change behavior, which allows for comparing the 7 studies. However, a generalization of the results for other interventions must be done with caution because some interventions also used other theories, such as self-regulation theories, as a framework for the educational content.

Although the restricted number of studies is a limitation, including other Web-based tailored interventions might have resulted in even higher program heterogeneity and would have made comparisons even more difficult and results (partly) dependent on program characteristics. Also Wienert and Kuhlmann [98] have determined that tailored interventions are difficult to compare, whereas the interventions included in this study were comparable because they all were based on the same theoretical background (the I-Change model), all 7 studies provided tailored feedback on social cognitive determinants from this model and provided feedback, and all 7 studies included the same program evaluation item. The comparison of interventions using other tailoring techniques than the interventions described in this study is difficult and access to the original data at the individual level would be necessary for further research and adequate analysis [99].

One of the strengths of this study is the access to 7 datasets (at participant level), which allowed us to conduct the analysis with the original data. Second, all studies used at least one identical item to assess the study evaluation which enables us to compare these studies. Finally, all studies had a large number of participants ranging from 1149 to 5055, which makes our results meaningful.

### Conclusion

This study showed that for 3 of 7 studies on computer-tailored interventions, participants with a lower educational level dropped out more often from follow-up measurements and tended to evaluate the interventions better compared to participants with a middle and higher educational level. However, the evaluation of the intervention did not predict participation or nonparticipation at follow-up. Based on our results, it is hard to say what other factors may play a role in dropout attrition from Web-based computer-tailored interventions. Further studies might evaluate different aspects of the intervention, besides only the participants’ grades, to find more relevant aspects of intervention evaluation.

Future studies should take high dropout among lower educated participants into consideration when developing strategies to decrease high dropout from Web-based computer-tailored interventions.

## Abbreviations

AEL: Alcohol-Everything within the Limit
COPD: chronic obstructive pulmonary disease
HSD: honestly significant difference
MHB: My Healthy Behavior
MYB: Master Your Breath
PAS: Personal Advice in Stopping smoking
RCT: randomized controlled trial
SQ4U: Stay Quit for You
STQ: Support to Quit
WIB: Weight in Balance
